# Some Aspects of Medical Research in the U.K.

**Published:** 1983-10

**Authors:** Brandon Lush

**Affiliations:** Consultant Physician in Geriatric Medicine, Frenchay and Manor Park Hospitals


### Some Aspects of Medical Research in the U.K.

**Brandon Lush, M.D., F.R.C.P.**
Consultant Physician in Geriatric Medicine, Frenchay and Manor Park Hospitals

### THE OBJECTIVES AND NATURE OF RESEARCH

The objective of research, medical or otherwise, is to add to existing knowledge. In practice, this means the systematic examination of present knowledge, the formulation of a hypothesis to test (this is a better word than prove, which has a second meaning less appropriate in this context) by careful observation and, usually, experiment – and analysis of the results. Research ranges in the medical field from simple bedside research and observations inseparable from the good practice of medicine to complicated experimental studies of cellular metabolism, of intracellular, and even intramolecular, structure.

At one end of the scale no special equipment is needed. It is open to any medical practitioner to add to knowledge by systematic observation of his (or her) patients, but he is unlikely to be able to add materially to the fundamental understanding of disease. This is not to belittle his contribution, for medical science must be based on clinical observation – a Sydenham must precede a Pasteur.

At the other end of the scale from the bedside, the frontiers of knowledge are advancing at an ever-increasing speed – so one has to travel further (i.e. to be properly trained) in order to reach them. Hence, training for medical research has become more important and this article provides a brief review of what has been done in this country to provide support for research and research training.

### SUPPORT FROM PUBLIC FUNDS

The concept that the promotion of medical research is a responsibility of the State is almost wholly a growth of the twentieth century – certainly as far as the U.K. is concerned. There were some public health investigations funded by the Poor Law and the General Board of Health in Victorian times – but these were on a very small scale and the General Board of Health was abolished in 1858.

The origins of the present Medical Research Council lie in Lloyd George's National Insurance Act of 1911. One of the provisions of that Act was to set aside the sum of one penny per head of the insured population per annum for research on the prevention of diseases of the insured. The intention was clearly to save money rather than for any altruistic motive. However, it was some two years before it occurred to anybody that some machinery had to be set up to spend the money! Hence it was not until 1913 that the first Medical Research Committee was set up under the Chairmanship of Lord Moulton, with distinguished medical membership (including Sir Thomas Clifford Allbutt, Sir Frederick Gowland Hopkins and Sir William Leishman). The money then available amounted to some £57,000 per annum.

The great scourge of the industrial population at that time was pulmonary tuberculosis and much important work on that subject was done under the auspices of the Committee in their early years. However, the 1914–18 war broke out soon after the initial investigations were begun so the Committee turned its attentions to war work and its funding was no longer tied to the 'penny per insured person per annum'. Among the many investigations initiated and carried out were those into hours of work, conditions (heating, lighting, ventilation, etc.) of work, health of munition workers and further studies on tuberculosis. At the beginning of that War, employers had the naïve idea that output per head increased arithmetically with the number of hours worked – but it was quickly shown that, not only did the output per hour fall if more than 8 hours were worked per day, but total output per day also fell. Thus a scientific basis was produced for rational and humane working conditions. It is sad to reflect that all this was forgotten at the beginning of the 1939–45 war – and it took some time to get the message over in appropriate quarters.

As the 1914–18 war was drawing to a close the Government of the day (through the Ministry of Reconstruction) took a look at what had been done

Dr. Lush worked for the M.R.C. for 22 years for the last 11 of which he was Principal Medical Officer. He was on the Grand Council and the Scientific Advisory Council of the British Empire Cancer Campaign. He was also Chairman of the Research Advisory Committee of the Mason Medical Research Foundation and was on the Anglo-French Bursary Committee of the Ciba Foundation. Editor.

and concluded that a public-funded organisation was necessary indefinitely to continue and extend the good work. There was much debate and discussion about the machinery for doing this. Some cabinet ministers felt strongly that a Medical Research Council should be set up within the Ministry of Health – but the late Lord Haldane and the late Viscount Addison felt strongly that a national research council should be quite independent of ministerial control and the whims of politicians – and they managed to get their way. Accordingly the U.K. Medical Research Council was set up by Royal Charter in 1920 under the aegis of a Committee (of Ministers) for Medical Research of the Privy Council, with the Lord President of the Council as Chairman. In fact, the Committee only met once and the Lord President of the day normally acted on its behalf.

This excellent system functioned magnificently until it was superseded in 1965 by bringing the Medical Research Council (and the other National Research Councils) under the wing of the newly created Department of Education and Science.

One of the great advantages of an M.R.C. independent of Government was that it could provide expert advice to Government quite independent of any form of political control – and it did so, with great effect, both formally and informally on such things as the effects of atomic radiations on human beings (a subject meriting a separate article) and on many other aspects of health policy. Although, in theory, the present M.R.C. is free from political control, cynics are liable to discredit any advice from a Government Department as necessarily biased. Nevertheless, the M.R.C. is still in a strong position to marshal the best available advice on medical topics and to make it available to Ministers in an appropriate form.

Returning to the question of the earlier years, the original Medical Research Committee regarded the setting up of a central research establishment as a matter of the highest priority but this did not prove possible until 1920, when the first National Institute for Medical Research was set up. It is fascinating to note that the original plans for the Institute in 1914 were that it should have 4 divisions – Bacteriology (under Sir Almwroth Wright;) Biochemistry and Pharmacology (under Sir Henry Dale); Applied Physiology (under Sir Leonard Hill) and Statistics (under Dr. J. Brownlee). Few at that time were aware of the importance that statistics were to play in medical research and the power-house of initial leading investigators would surely have done great things (as they did later separately – but never had the chance to collaborate closely as planned).

Sir Henry Dale became the first Director of the National Institute for Medical Research, (N.I.M.R.), and turned it into a breeding ground for Professors in many subjects. While much of its success was due to brilliant leadership, unquestionably it was the fact that it was so much better facilitated than University Departments in those days – and free of formal teaching and examining duties which helped it to become a world centre of medical research.

From the outset the M.R.C. were aware that medical research was constantly changing and that flexibility was absolutely essential. Hence they adopted the policy of setting up 'Units', usually in University Departments, under hand-picked leading investigators. The life of the Units was essentially that of its Directors working life and units were disbanded if and when a Director left (and if no 'Crown Prince' had emerged). Even at N.I.M.R. new Divisions were created from time to time and old ones disbanded or split.

The results of these policies lead to many major advances in medical and biological sciences, including, in effect, the creation of new branches of science – the most famous at present probably being molecular biology. Many whole-time M.R.C. staff were awarded Nobel Prizes – one of them twice (Frederick Sanger). Space does not permit going into further detail.

In addition to supporting their own staff, the M.R.C. have always been a grant-giving body and have supported literally thousands of investigations, which they regarded as of 'special timeliness and promise', primarily (but not exclusively) in Universities. Finally, they have also initiated and continued many training programmes – at all appropriate levels – for those wishing to undertake medical research – not necessarily as a career.

### HEALTH DEPARTMENTS

Until recently the Ministry of Health and its successor, the Department of Health and Social Security, had played only a minor role in supporting research. However, the successive Chief Medical Officers all had a small annual sum, known as 'the C.M.O.'s Fund', which they could use for the support of research directly related to the needs of the Ministry. However, for most research the Ministry used to look to the M.R.C. Regrettably, the feeling grew in the Ministry and the later D.H.S.S., that the M.R.C. was not as helpful in that regard as it should have been. Inevitably there are two sides to this unhappy saga which would take too long to describe. Suffice it to say that the end result from 1972 onwards was that the so-called Rothschild 'Consumer/Contractor relationship' was introduced whereby the D.H.S.S. was supposed to control, in effect, a quarter of the M.R.C.'s research programme. This misguided concept never really took off and an unhappy period was terminated by mutual agreement in 1982.

The 'in-house' investigations of the Health Departments were, nevertheless, most useful.

### PRIVATE FUNDS

Philanthropy has always been a major factor in the British scene – and medical research is no exception. Everyone is familiar with the large Cancer Research Organisations – but there are literally scores of other medical research charities in the U.K. – and they are playing an ever-increasing role in the support of medical research. Between them they currently have some £80,000,000 p.a. to disburse.

So much for a potted history of the evolution of support for medical research in the U.K. – but how does one obtain some of the millions of pounds available annually?

The first point is to have a clear idea of exactly what one wants to do – and what it is likely to achieve. The commonest question grant-awarding bodies ask themselves when considering applications is, to put it crudely, 'so what?'. In other words, what significant advance in medical knowledge is likely to result when the research project is completed. Far too many proposed investigations involve the collection of vast amounts of data in the hope that a computer will subsequently analyze and produce significant useful new information. It seldom, if ever, does unless the data has been collected to a clear plan and that there is a hypothesis to test.

Having decided on a good investigation, the next question is 'what is the appropriate body to approach for support?' – for, just as there are horses for courses, so there are particular projects for particular grant-giving bodies and *vice versa*. One is simply wasting time in approaching an inappropriate potential sponsor, so it is worth spending time in studying information on the scope of several before deciding on one ('blunderbuss' applications to several grant-giving bodies at once are frowned upon).

The administrative overheads in processing grant applications in the major research organisations are now so large that it is pointless to apply to them for small sums of money – only for substantial three-year projects. Information on all aspects of research grants (including 5-year so-called programme grants) and research training schemes is obtainable from the Medical Research Council, 20, Park Crescent, London W1N 4AL.

The Wellcome Foundation, Park Square West, Regents Park, London W1, covers a narrower range than the M.R.C. (which, to all intents and purposes, covers any medical research). The Foundation is particularly interested in tropical medicine and the history of medicine and they run a number of schemes intended to fill gaps left by other organisations. They are particularly interested in cross-disciplinary research projects.

The remaining charitable organisations, varying enormously in size and range, are listed in the annual Handbook of the Association of Medical Research Charities (obtainable from the A.C.M.R., c/o British Heart Foundation, 102, Gloucester Place, London, W1H 4DH).

Coming down slightly in scale, each Region is provided with research funds intended primarily for clinical research 'inseparable from the good practise of medicine' and details can be obtained from the Regional Medical Officer.

Finally, most hospitals have some funds which can be used for approved research – but the amounts available are small – and often used up early in the financial year.

(Those wishing more information on the history of the MRC should refer to 'Half a Century of Medical Research' by Sir A. Landsborough Thomson, HMSO, London, 1973.)

